# Prevalence of methicillin resistance and superantigenic toxins in *Staphylococcus aureus* strains isolated from patients with cancer

**DOI:** 10.1186/s12866-021-02319-7

**Published:** 2021-09-29

**Authors:** Effat Abbasi Montazeri, Azar Dokht Khosravi, Saeedeh Khazaei, Ali Sabbagh

**Affiliations:** 1 Infectious and Tropical Diseases Research Center, Health Research Institute, Ahvaz Jundishapur University of Medical Sciences, Ahvaz, Iran; 2 Department of Microbiology, Faculty of Medicine, Ahvaz Jundishapur University of Medical Sciences, Ahvaz, Iran; 3 Thalassemia and Hemoglobinopathy Research Center, Research Institute of Health, Ahvaz Jundishapur University of Medical Sciences, Ahvaz, Iran

**Keywords:** Cancer patients, Methicillin-resistance *Staphylococcus aureus*, MRSA, Spa typing, Superantigenic toxins

## Abstract

**Background:**

This study aimed to determine the frequency of methicillin-resistant *Staphylococcus aureus* (MRSA), antibiotic resistance patterns, superantigenic toxins profile, and clonality of this pathogen in patients with cancer.

**Results:**

In total, 79 (25.7%) isolates were confirmed as *Staphylococcus* species, from which 38 (48.1%) isolates were *S. aureus,* and 29 (76.3%) isolates were confirmed as MRSA. The highest resistance in MRSA strains was seen against ciprofloxacin (86.2%) and erythromycin (82.8%). Teicoplanin, and linezolid were the most effective antibiotics. From all MRSA isolates, 3 strains (10.3%) were resistant to vancomycin with minimum inhibitory concentration values of 128 μg/ml. The prevalence of superantigenic toxins *genes* was as follows: *pvl* (10.5%), *tsst-1* (36.8%), *etA* (23.7%), and *etB* (23.7%). The t14870 *spa* type with frequency of 39.5% was the most prevalent clone type circulating in the cancer patients.

**Conclusions:**

This study showed the circulating of *spa* t14870 as the most predominant MRSA clone in cancer patients of southwest Iran. Also, a diverse antibiotic resistance pattern and toxin profiles were seen among MRSA isolates.

## Background

*Staphylococcus aureus* is one of the most important pathogens in both hospital- and community- acquired infections with significant patient morbidity and mortality rate worldwide [1]. *S. aureus* as a potential pathogen causes various infections including superficial and deep skin infections, soft tissue infections, bacteremia, endocarditis, osteomyelitis, pneumonia, toxic- shock syndrome, and staphylococcal scaled skin syndrome [2]. Over the past several decades, following the introduction of antibiotics, emergence and dissemination of methicillin resistant *S. aureus* (MRSA) remains a challenging public health issue [3]. MRSA strains characterized by the presence of the *mec*A gene, which is located on the staphylococcal chromosomal cassette mec (SCC*mec* type I - XI) as a mobile genetic element and encoding low-affinity penicillin binding protein (PBP2-a) have resistance to multiple antibiotics including penicillin and other beta-lactams [1, 4].

Both MRSA and methicillin-sensitive *S. aureus* (MSSA) strains produce several virulence factors and toxins that have a significant role in the pathogenicity of the diseases and notably that some of them have superantigenic properties [5]. *S. aureus* superantigens (SAgs) are known as the most potent T cell mitogens. They stimulate large subpopulations of T lymphocytes via cross-linking their T cell receptor with major histocompatibility complex class-II molecules (MHC-II) on antigen presenting cells, resulting in T cell and B cell proliferation and massive cytokine release which may lead to systemic shock [6]. To date, more than 26 different superantigens have been described in the *S. aureus* species; comprising the toxic shock syndrome toxin (TSST-1), exfoliative toxins (ETs), Panton-Valentine leukocidin (PVL), 11 staphylococcal enterotoxins (SEs), 14 enterotoxin-like proteins (SEls) as well as staphylococcal protein A (spA); that is the only known B cell superantigen produced by *S. aureus* [6, 7].

The capability of MRSA to cause a broad range of human diseases is based on combination of host factors and bacterial virulence factors [2]. There are several risk factors that cause patients at higher risk for acquisition infections with MRSA including age, length of hospitalization, initial antibiotic treatment, severe underlying disease, surgery and compromised host immunity [2, 6]. Particularly, cancer patients due to impaired cellular immunity, are very prone to serious infections with significant complications particularly with multidrug resistant bacteria (MDRB), and they have multiple predisposing factors that increase the risk of infection; including chemotherapy, radiation therapy, surgery, stem cell transplantation, bone marrow transplantation [8].

Emergence of antimicrobial resistance among MRSA associated with infections in cancer patients is particular concern in recent years. In addition, only limited data are available for the MRSA distribution, antibiotic resistance patterns, and prognosis of these infections in oncological patients [9]. Consequently, newer therapeutic approaches, required to be developed based on local epidemiologic and susceptibility/resistance data for infection prevention, control, and antimicrobial stewardship are important in the overall management of infections in cancer patients [10].

Therefore, this research was conducted for the first time on cancer patients hospitalized in Ahvaz Oncology Center to determine the resistance patterns, and superantigenic toxins profiles (*etA*, *etB*, *tsst-1*, and *pvl*) in MRSA isolates collected from cancer patients. Also, the profile of *Staphylococcus aureus* protein A (*spa*) types was investigated in the isolates.

## Methods

### Sampling and data collection

This cross-sectional study was performed during 16 months from February 2018 to March 2019. In total, 307 bacterial isolates were obtained from various clinical specimens of hospitalized cancer patients referred to Shahid Baghaei educational hospital, one of the main referral centers for oncologic disorders in Ahvaz, Khuzestan Province, southwest of Iran. These isolates were recovered from blood, wound, tracheal tube secretions, eyes, ears, urine, abscess, and cerebrospinal fluid (CSF). The collected isolates were transferred to the Department of Microbiology of Ahvaz Jundishapur University of Medical Sciences. These isolates were identified by conventional microbiological methods. In brief, bacterial isolates were subcultured on Blood Agar (Merck, Germany) plates and incubated at 37°C for 24 h. Finally, they were identified as staphylococci strains using phenotypic laboratory methods such as colony morphology, Gram stain, and conventional biochemical tests including catalase test, mannitol fermentation, DNase production, and plasma coagulase reaction [11]. The *S. aureus* ATCC 29213 strain was used as the reference strain. All phenotypically identified staphylococci were stored in Trypticase Soy Broth (TSB; Merck, Germany) containing 20% glycerol and were kept at −70°C for further molecular investigation.

### Confirmation of *S. aureus* and coagulase-negative staphylococci (CoNS) isolates by polymerase chain reaction (PCR)

The *16S rRNA* (specifically detecting *Staphylococcus* species) and *nuc* (distinguishing *S. aureus* from CoNS) genes amplification was done by a multiplex-polymerase chain reaction (M-PCR) using sets of designed primers as previously described (Table 1) [12].

**Table 1:** The primers sequences and the products sizes of the studied genes used in this study

Target	Gene	Primer sequence (5′-3′)	Annealing Tm	Size of product (bp)	Reference
*16 s rRNA*	*16 s rRNA*	F: AACTCTGTTATTAGGGAAGAACA R: CCACCTTCCTCCGGTTTGTCACC	59 C ° for 1 min	756	[12]
*nuc*	*nuc*	F: GCGATTGATGGTGATACGGTT R: AGCCAAGCCTTGACGAACTAAAGC	59 C ° for 1 min	279	[12]
*mecA*	*mecA*	F: GTAGAAATGACTGAACGTCCGATAA R: CCAATTCCACATTGTTTCGGTCTAA	58C ° for 1 min	310	[14]
*Toxin genes*	*etB*	F: ACAAGCAAAAGAATACAGCG R: GTTTTTGGCTGCTTCTCTTG	53C° for 45	226	[4]
	*etA*	F: GCAGGTGTTGATTTAGCATT R: AGATGTCCCTATTTTTGCTG	53C° for 45	93	[4]
	*pvl*	F: ATCATTAGGTAAAATGTCTGGACATGATCC R: GCATCAAGTGTATTGGATAGCAAAAGC	57 C ° for 30 s	433	[16, 33]
	*tsst-1*	F: TTATCGTAAGCCCTTTGTTG R: TAAAGGTAGTTCTATTGGAGTAGG	53C° for 45	398	[4]

### Methicillin-resistant *S. aureus* and methicillin-resistant coagulase-negative staphylococci (MRCoNS) screening

MRSA and MRCoNS isolates were identified with cefoxitin disc (30 μg) on Mueller Hinton Agar (MHA, Merck, Germany) plates in accordance with the Clinical and Laboratory Standards Institute (CLSI) guidelines [13]. Finally, the resistant isolates were confirmed as MRSA and MRCoNS using PCR for amplification of *mec*A gene according to previous study (Table 1) [14]. The *S. aureus* ATCC 33591 and ATCC 25923 were used as the positive and negative controls for the *mec*A gene, respectively.

### Antimicrobial susceptibility testing of *Staphylococcus* isolates

*In vitro* antimicrobial susceptibility testing determined by Kirby-Bauer disk diffusion according to the CLSI guidelines [13]. The 10 antimicrobial disks tested included: cefoxitin (FOX, 30 μg), clindamycin (CD, 2 μg), ciprofloxacin (CIP, 5 μg), cotrimaxazole (TS, 25 μg), rifampicin (RP, 5 μg), teicoplanin (TEC, 30 μg), tetracycline (T, 10 μg), erythromycin (E, 15 μg), quinupristin/dalfopristin (SYN, 15 μg), and linezolid (LZD, 30 μg) [MAST Diagnostics, Merseyside UK]*.*

.Also, vancomycin susceptibility testing was performed by vancomycin agar screen method with brain heart infusion agar containing 6 μg/ml. Finally, the minimum inhibitory concentration (MIC) of vancomycin was determined with the broth microdilution method according to CLSI 2016 instructions [13]. The vancomycin powder was purchased from Sigma (USA) Company. *S. aureus* ATCC 29213 and ATCC 33591 were used as methicillin-sensitive and resistant control strains, respectively.

### Detection of superantigenic toxin genes (*etA, etA, tsst-1,* and *pvl*)

#### Extraction of genomic DNA

Genomic DNA was extracted from pure colonies of MRSA strains using the boiling method as described previously [15]. A Biophotometer (Eppendorf, Germany) at 260 nm, was used to measure the concentration of DNA. The extracted DNA were stored at -20 ° C for further molecular investigation.

### PCR protocol

Several PCR amplifications were carried out for the detection of superantigenic toxin genes using specific primers based on *etA, etB, tsst-1, and pvl* genes as previously described [4, 16]. The sequences of primers are listed in Table 1. The synthesis of primers was carried out by the TAG Copenhagen Company, Denmark. The M-PCR was optimized on an Eppendorf thermocycler (Roche Co., Germany), in a final volume of 25 μl containing 12.5 μl of PCR Master Mix, 0.4 mM (1 μl) of each forward and reverse primers, 5 μl of DNA template, and 9.5 μl of nuclease-free water. The amplicons products were analyzed using an ultraviolet gel documentation device (Protein Simple, San Jose, CA, USA), after loading in the gel electrophoresis tank on a 1.5% agarose gel (w/v) with 2 μl safe stain (Sinaclon Co., Tehran, Iran).

### Staphylococcal protein a (*spa*) typing

The *spa* typing was performed for MRSA and MSSA strains with some modification by PCR as previously described [17]. The polymorphic X regions of the *spa* gene from the extracted chromosomal DNAs were amplified using the primers spa 1113F (5′-TAAAGACGATCCTTCGGTGAGC-3′) and spa-1514R (5′-CAGCAGTAGTGCCGTTTGCTT-3′). The variable X region of the *spa* gene was amplified in a 25 μl reaction volume and optimized on an Eppendorf thermo-cycler (Roche Co., Berlin, Germany). The PCR mixture contained 12.5 μl 1.5×TaqMaster Mix, 2 μl of each spa primers (10 μM), 1 μl chromosomal DNA template and 7.5 μl double distilled water. The PCR conditions were as follow: initial denaturation at 94 °C for 5 min, followed by 30 cycles of denaturation at 94 °C for 30s, annealing at 60 °C for 60s, and extension at 72 °C for 30s, and a final extension at 72 °C for 10 min. The amplicons products were subjected to DNA sequencing for both strands by Macrogen Seoul, South Korea). The sequences obtained were edited using Chromas software (version 1.45, Australia). The Ridom Spa Server database (http://www.spaserver.ridom.de) (Ridom, Wurzburg, Germany) was used to assign the edited sequences to particular *spa* types [17].

### Statistical analysis

Data were analyzed using SPSS software version 25 (IBM Corporation, Armonk, NY, USA). The Chi-square test and Fisher’s exact test was used for the association between the studied genes and the presence of MRSA strains. A *p*-value < 0.05 was considered statistically significant.

## Result

### Studied isolates

In current study, out of the 307 clinical bacterial isolates, 79 (25.7%) isolates were confirmed as *Staphylococcus* species and the rest of the 228 isolates (74.3%) were Gram-negative bacteria based on culture and standard biochemical criteria. All *Staphylococcus* isolates were evaluated for the presence of *nuc* and *16S rRNA* genes to confirm and select the *S. aureus* isolates. According to PCR analysis, from 79 *Staphylococcus* isolates, 38 (48.1 %) were molecularly confirmed as *S. aureus* by the presence of *16S rRNA* and *nuc* genes, while the remaining 41 isolates (51.9 %) were different coagulase-negative staphylococci (CoNS) species. The isolates were collected from 35 male (44.3 %) and 44 female patients (55.7 %), respectively. There was no statistically significant difference in gender distribution (*P* = 0.75). The total distribution of 79 *Staphylococcus* isolates in various types of clinical specimens was as follows: blood 64 (81 %), urine 7 (8.9 %), wound 5 (6.3 %) and catheters 3 (3.8 %). The distribution of isolated *Staphylococcus* species according to the hospital wards was as follows: hematology intensive care unit (HICU) 31 (39.3 %), transplantation and bone marrow transplantation (BMT) 12 (15.2%), pediatric 17 (21.5 %), women 11 (13.9 %), men 5 (6.3 %), and thalassemia unit 3 (3.8 %).

### MRSA and MRCoNS screening

The results of cefoxitin test and *mec*A PCR amplification confirmed 76.3% (29/38) of *S. aureus* strains as MRSA, while the 9 (23.7 %) remaining isolates were considered as methicillin-sensitive *S. aureus* (MSSA). Also, among the CoNS species, 65.9% (27/41) and 34.1% (14/41) of isolates were confirmed as MRCoNS and methicillin-sensitive CoNS (MSCoNS), respectively. The both methods (cefoxitin test and mecA PCR) showed similar results. The prevalence rate of MRSA in different clinical specimens was as follows: blood samples (n=22/29, 75.9%), catheters and urine each one (n =3/29, 10.3%), and wound (n=1/29, 3.5%). The most frequency of MRSA isolates (75.9%) were seen in blood samples. The highest prevalence of MRSA isolates was seen in HICU with 41.5% (n=12/29) rates. Although the prevalence of MRSA strains was higher in blood culture samples (91.7 %, n = 11/12), but no significant difference was observed in the frequency of strains in different cancer patients admitted to HICU (*P* > 0.05). The frequency of MRSA in other wards was as follows: pediatric and BMT: (17.2%, n=5/29 for each wards), women and men wards (10.3%, n=3/29 for each wards), and thalassemia unit (3.5%, n=1/29).

### Antimicrobial susceptibility testing of *S. aureus* and CoNS

**Fig. 1:**
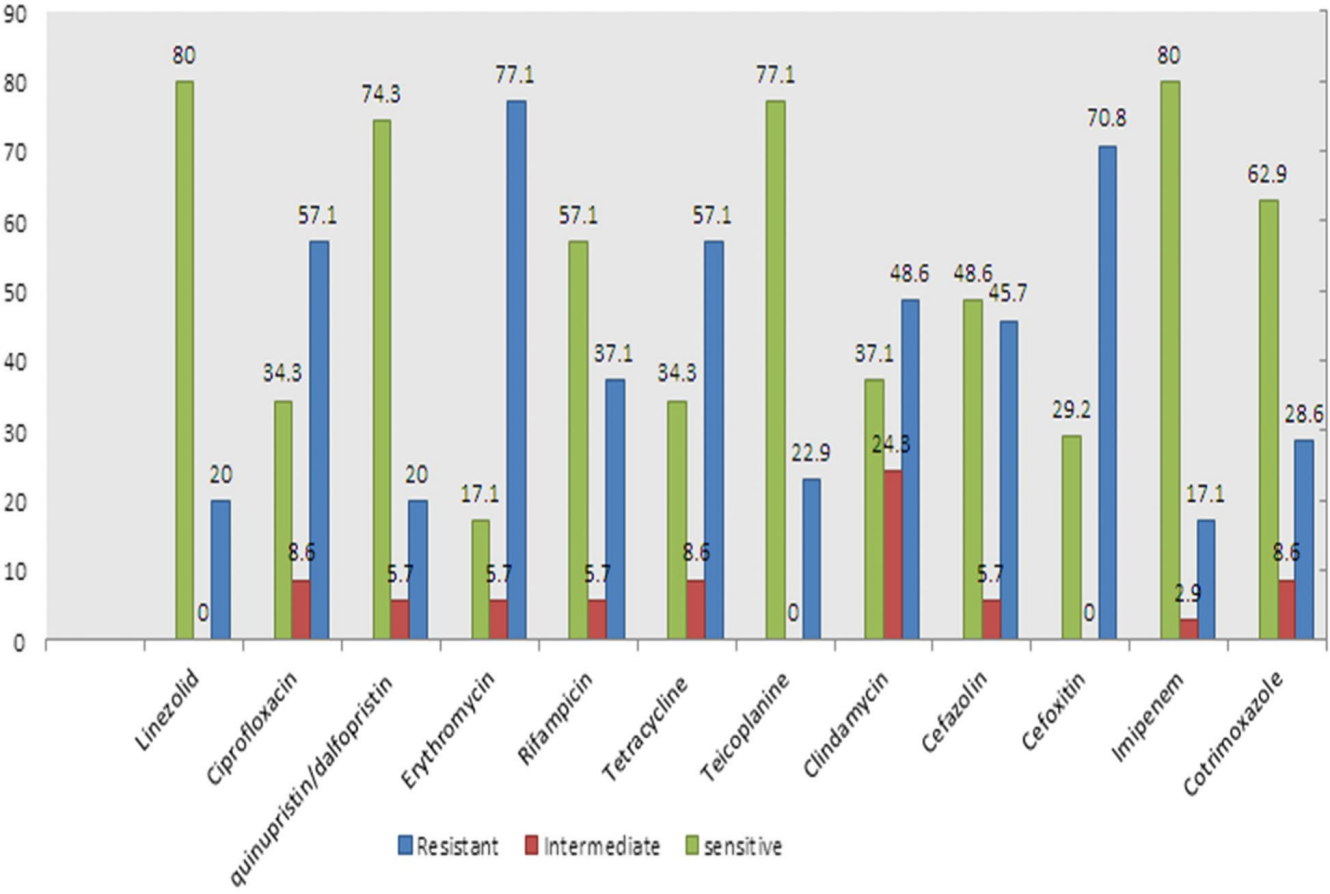
Antibiotic resistance rates (%) of all *Staphylococcus* isolates

The susceptibility patterns of total 79 *Staphylococcus* isolates demonstrated highly resistant to erythromycin (77.1%) and cefoxitin (70.8%). The linezolid with susceptibility rate of 80%, was the most effective antibiotic. The resistance rates of all *Staphylococcus* isolates are shown in Fig. 1.

The susceptibility patterns of MRSA strains against 10 various antibiotics are presented in Table 2. A high rate of resistance was detected against ciprofloxacin (n=25/29, 86.2 %), erythromycin (n=24/29, 82.8 %), and clindamycin (n=20/29, 69 %). The resistance to clindamycin was constitutive phenotype and no inducible phenomenon was detected. MRSA isolates had the lowest prevalence of resistance against teicoplanin and linezolid (n=3/29, 10.4% for each one). From a total of 29 MRSA isolates, 3 strains (10.3 %) were resistant to vancomycin using vancomycin agar screen test. Subsequently, the broth microdilution assay confirmed that these isolates had MIC values of 128 μg/ml (Table 3). All VRSA strains were isolated from blood culture of hospitalized patients in the ICU. They were susceptible to linezolid. The resistance rates of different antibiotics against CoNS isolates were as follows: erythromycin 90.2%, clindamycin 70.7 %, cefoxitin 65.9 %, tetracycline 48.2 %, rifampicin 46.3 %, ciprofloxacin 46.3 %, cotrimoxazole 39.1 %, teicoplanin 12.2 %, and linezolid 9.8%.

**Table 2:** Antimicrobial susceptibility of MRSA strains isolated from cancer patients in this study

Antimicrobial category	Antimicrobial agents	Concentration of disk (μg)	Sensitive n (%)	Intermediate n (%)	Resistant n (%)
**Anti-staphylococcal β-lactams (or cephamycins)**	Cefoxitin (FOX)	30	–	–	29 (100%)
**Folate pathway inhibitors**	Cotrimoxazole (TS)	25	17 (58.6%)	2 (6.9%)	10 (34.5%)
**Lincosamides**	Clindamycin (CD)	2	7 (24.2%)	2 (6.8%)	20 (69%)
**Glycopeptides**	Teicoplanin (TEC)	30	25 (86.2%)	1 (3.4%)	3 (10.4%)
**Tetracyclines**	Tetracycline (TE)	10	12 (41.4%)	–	17 (58.6%)
**Ansamycins**	Rifampicin (RP)	5	12 (41.4%)	2 (6.8%)	15 (51.8%)
**Macrolides**	Erythromycin (E)	15	4 (13.8%)	1 (3.4%)	24 (82.8%)
**Streptogramins**	Quinupristin/dalfopristin (SYN)	15	21 (72.4%)	–	8 (27.6%)
**Fluoroquinolones**	Ciprofloxacin (CIP)	5	4 (13.8%)	–	25 (86.2%)
**Oxazolidinones**	Linezolid (LZD)	30	26 (89.6%)	–	3 (10.4%)

**Table 3:** The minimum inhibitory concentration (MIC) of vancomycin of studied MRSA isolates with broth microdilution

MIC (μg /ml)	128	64	32	16	8	4	2	1	0.5	0.25
n (%)	3 (10.3)	0 (0.0)	0 (0.0)	0 (0.0)	0 (0.0)	0 (0.0)	0 (0.0)	2 (6.9)	4 (13.8)	20 (69)

### Detection of *etA, etB, tsst-1*, and *pvl* genes

A total of 38 *S. aureus* isolates were subjected to superantigenic toxin genes *etA, etB, tsst-1, and pvl* analysis. Twenty isolates (n=20/38, 52.6%) harbored at least one superantigenic toxin genes. Distribution of these genes was as follows: 36.8% (n=14/38), 23.7% (n=9/38), 23.7% (n=9/38), and 10.5% (n=4/38) for *tsst-1*, *etA*, *etB*, and *pvl*, respectively. Finally, 17 (n=17/29, 58.6%) of MRSA and 3 (n=3/9, 33.3%) of MSSA isolates produced one of these toxins. Also, 13.8 % of MRSA isolates, (n=4/29) had 3 *tsst-1*, *etA* and *etB* genes, simultaneously. Also, prevalence rate of concurrent *tsst-1* and *etA* genes in MRSA isolates were 3.4 % (n=1/29). The *tsst-1* and *etB* genes were simultaneously detected in 10.3 % (n=3/29). Also, no association was found among the presence of superantigenic toxins and antibiotic resistance (data not shown).

### *Spa* typing

According to *spa* typing in this study, 12 different *spa* types were observed among the 38 *S. aureus* strains. The *spa* type t14870 was the most common type (39.5%) among the isolates. According to statistical tests, there was no significant difference in the distribution of *spa* types with different hospital wards and clinical specimens. Most of the samples in this study were blood and the most common type t14870 was isolated from these samples (n=13/38, 34.2%). There was no significant relationship between the distribution of different types and sample type (*P* = 0. 72). Also, among the isolates that were positive for *pvl, tsst-1, etA, etB,* and *mec* genes, the type t14870 was the most prevalent compared to all other types. Two VRSA isolates had *spa* type t14870 and one of them had *spa* type t030. The distribution of different *spa* types among *Staphylococcus* isolates is shown in Table 4.

**Table 4:** Distribution of *spa* types among MRSA and MSSA isolates in this study

*Spa* type	Frequency	Percentage (%)	MRSA	MSSA
t14870	15	39.5%	13	2
t1814	1	2.6%	–	1
tT1149	1	2.6%	1	–
t5598	1	2.6%	1	–
t386	3	7.8%	3	–
t030	2	5.2%	2	–
t711	1	2.6%	1	–
t1671	2	5.2%	–	2
t003	1	2.6%	–	1
t701	1	2.6%	1	–
t605	1	2.6%	–	1
t421	1	2.6%	–	1
Not type	8	21%	7	1
Total	38	100%	29	9

## Discussion

The circulation of MRSA in health systems and community leads to increase of the costs of antibiotic therapy and limits the cure options. During the recent decades, there have been extensive studies on the resistance pattern of MRSA strains [18]. It has been observed that some MRSA clones spread very rapidly in different regions [19]. Hence, the determination of the regional pattern of resistance is helpful in selecting the appropriate drug. The current study was the first that performed to investigate the resistance patterns and molecular epidemiology of MRSA common clones among cancer patients in the Ahvaz city, southwest region of Iran. So far, no epidemiologic data about MRSA strains and their different clone types have been reported in cancer patients in aforesaid area.

Recent data from several cancer centers/organizations indicated the CoNS strains as the predominant Gram-positive pathogens isolated from various infections in cancer patients [20, 21]. The current research in agreement with previous studies showed the CoNS species as the most common Gram-positive bacteria with frequency of 51.9 % (n = 41/79). However, the total frequency rate of CoNS with 13.4 % (n = 41/307) was much lower than a study by Garcia-Vidal et al. [21] from Spain who reported an incidence of 35.7% for these species. In another study by Islas-Muñoz et al. [20] from Mexico, the frequency rate of CONS isolates (11 %) was almost close to our result. CoNS isolates showed the most resistance rates (more than 60 %) against erythromycin and clindamycin. Similar to the current research, Ghadiri et al. [22] from Tehran, Iran, and Fahim et al. [23] from Egypt reported a high resistance rate against erythromycin and clindamycin.

The current study showed the total prevalence of 9.4 % (n = 29/307) for MRSA in the cancer patients that was lower than several previous studies from Japan 77.8% [24], Egypt 70% [25], Libya 35.5% [26], France 44.4% [27], and Kerman city in Iran 28% [28]. Besides, the total prevalence of 8.8 % for MRCoNS isolates in this study was lower than the total incidence rate of a study by Fentie et al. [29] who revealed a prevalence of 11.6 %. These dissimilarities of the results of various studies may be due to the differences in studied sample size, epidemiology of the study area, the bacterial detection methods, and the type of studied samples. In this research, the prevalence of MRSA strains in HICU ward (41.5%) was higher than other wards that was in line with the study by Srinivasan et al. [30] who reported the highest frequency of MRSA in the ICU ward (15%) of the hospital.

In the current research, the results of disk diffusion method showed the highest resistance rate of MRSA isolates against ciprofloxacin (86.2%), erythromycin (82.8%), and clindamycin (69%). The resistance rate of > 50 % against ciprofloxacin, erythromycin, and clindamycin was in parallel with the results of Bai et al. [31] from China. Also, in their study the resistance rate of cotrimoxazole (37%) was almost close to our result. In our studied region, the lowest resistance rates were seen against teicoplanin and linezolid that was in line with several previous reports from Iran and other countries [31–34]. In this study, 3 (10.3 %) MRSA isolates were resistant to vancomycin. The overall vancomycin-resistant *Staphylococcus aureus* (VRSA) prevalence was 7.9 % (3/38) that was meaningly different from its global occurrence (2.4 %) [35]. More frequent use of vancomycin for treatment of MRSA infections, improved diagnostic methods, inadequate surveillance for drug-resistant strains, a possible change in the vancomycin-resistance breakpoints since 2006, and the increased use of antibiotics as a food supplement are all possible reasons for the emergence or detection of more VRSA strains in recent years [35].

Another notable finding in the current study was the high resistance rate of MRSA to ciprofloxacin, which may be due to the increased use of fluoroquinolone antibiotics as prophylaxis to prevent infection in neutropenic cancer patients [36]. Overall, the results emphasize that a new surveillance program should be designed to determine the correct pattern of antibiotic use for cancer patients in the study area. Also, the indiscriminate use of ineffective antibiotic classes such as quinolones that may lead to *Clostridioides difficile* selection should be avoided [37].

To date, very little information is available on the prevalence of *S. aureus* superantigen genes in cancer patients, especially in Iran country. The findings of the current experiment showed that 58.6 % of MRSA isolates had at least one toxin genes that was much lower than a study by Jaradat et al. [38] from Jordan who showed 100 % of isolates harbored at least one toxin gene. In this study the prevalence of *pvl*, *tsst-1*, *etA*, and *etB* superantigenic toxins in *S. aureus* strains were 10.5 %, 36.8 %, 23.7 %, and 23.7 % respectively. Campo et al. [39] from USA reported frequency rate of 40 % for *pvl* gene in MRSA strains in cancer patients. The frequency rates of *pvl* gene in other studies that were performed in non-cancer patients were as follows: Iran 26.3 % [4], Jordan 100 % [38], China 45.2 % [40], and Germany 6.2 % [41]. Also, the prevalence of the *tsst-1* gene in the previous studies in non-cancer patients was as follows: Iran 32.6 % [4], Jordan 29.5 % [38], and Greece 3.5 % [42]. The frequency rate of *etA* gene in this research was lower than a report by Salas et al. [43] from Spain (71.3 %) and Jaradat et al. [38] from Jordan (42%). However, the detection rate of *etB* gene was so higher than Jaradat et al. [38] from Jordan and Horváth et al. [44] from Hungary who reported 0.0 % and 1.3 % of prevalence for aforesaid gene, respectively. While the frequency rates of two *etA* and *etB* genes were similar in our research, some studies reported the more frequency of *etA* compared to *etB* [43, 45]. According to this study, the incidence of superantigen genes showed that these virulence factors play an efficient role in MRSA infections in the cancer patients.

In this study, 12 different *spa* types were found that t14870, t386, t030, and t1671 with frequency rates of 39.5 %, 7.8%, 5.2%, and 5.2% were the most prevalent types, respectively. There was no significant association between patterns of *spa* gene with different parts of hospital and clinical samples (*P* value > 0.05). In Iran, many studies have been done on the typing of *S. aureus* strains. In a previous study from Tehran, Iran the t030 and t037 *spa* types accounted for 43% of all 11 different detected types [46]. In contrast to previous study from southwest of Iran by Hashemizadeh et al. [47] who showed the t030 as the most prevalent *spa* type among *S. aureus* isolates, this study revealed the t14870 as the most circulating *spa* type in the cancer patients. These discrepancies may be due to the study population, the type of sample being tested, and the pattern of used antibiotics that has enabled species with greater adaptability to form the dominant population. All different *spa* types that were detected in this research have been reported previously from Iran and other countries [17, 46–48]. Although, the *spa* type t14870 was previously reported in Iran and other regions, the current study was the first report for this *spa* type in cancer patients in southwest Iran. In line with the current study, Faramand et al. [49] reported the t14870 as the most prevalent *spa* from *S. aureus* isolated from raw beef and chicken meat samples.

## Conclusions

This study showed a high rate of MRSA circulation in cancer patients from southwest of Iran. Also, the *tsst-1* and *etA* were the most prevalent virulence genes. This study indicated a high prevalence of t14870 MRSA isolates in cancer patients from southwest of Iran. Moreover, this investigation showed a relatively high resistance rate against ciprofloxacin, erythromycin, and clindamycin among MRSA isolates and these antibiotics should be used with extreme caution based on the results of antibiogram tests. It is recommended that regular monitoring programs be among the priorities of health policy makers in order to reduce the faster spread of resistant strains in the population of cancer patients and reduce the resulting mortality.

## Data Availability

Not applicable.

## Abbreviations

BMT: Bone Marrow Tranplantation
CSF: Cerebrospinal Fluid
MRCoNS: Methicillin-Resistant Coagulase-Negative Staphylococci
MRSA: MSSA: Methicillin-Sensitive *Staphylococcus aureus*; Methicillin-Resistance *Staphylococcus aureus*
PVL: Panton-Valentine leukocidin
PCR: Polymerase Chain Reaction
*spa*: Staphylococcal protein A
